# No effects of a 12-week supervised exercise therapy program on gait in patients with mild to moderate osteoarthritis: a secondary analysis of a randomized trial

**DOI:** 10.1186/s12952-015-0023-y

**Published:** 2015-03-05

**Authors:** Ingrid Eitzen, Linda Fernandes, Lars Nordsletten, May Arna Risberg

**Affiliations:** Norwegian research Centre for Active Rehabilitation, Oslo, Norway; Orthopaedic Department, Oslo University Hospital, Oslo, Norway; Department of Orthopaedic Surgery and Traumatology, Institute of Clinical research, University of Southern Denmark, Odense, Denmark; University of Oslo, Oslo, Norway; Norwegian School of Sport Sciences, Oslo, Norway; Postal address: Orthopaedic Department, Oslo University Hospital, Kirkeveien 166, Bygg 72, 2. Etg., 0407 Oslo, Norway

**Keywords:** Hip osteoarthritis, Gait analysis, Biomechanics, Exercise therapy

## Abstract

**Background:**

It is unknown whether gait biomechanics in hip osteoarthritis patients with mild to moderate symptoms change following exercise therapy interventions. The aim of the present study was to compare stance phase gait characteristics in hip osteoarthritis patients with mild to moderate symptoms participating in a randomized trial with two different interventions; patient education only or patient education followed by a 12-week supervised exercise therapy program.

**Results:**

The study was conducted as a secondary analysis of a single-blinded randomized controlled trial. Patients aged 40 to 80 years, with hip osteoarthritis verified from self-reported pain and radiographic changes, were included. The final material comprised 23 patients (10 males/13 females, mean (SD) age 58.2 (10.02) years) in the patient education only group, and 22 patients (9 males/13 females, mean (SD) age 60.2 (9.49) years) in the patient education + exercise therapy group. Three-dimensional gait analysis was conducted at baseline and at four month follow-up. Sagittal and frontal plane joint angle displacement and external joint moments of the hip, knee and ankle were compared from a one-way analysis of covariance between the groups at follow-up, with baseline values as covariates (p < 0.05). No group differences were observed at the four-month follow-up in gait velocity, joint angle displacement, or moments. As the compliance in the exercise therapy group was inadequate, we calculated possible associations between the number of completed exercise sessions and change in each of the kinematic or kinetic variables. Associations were weak to neglible. Thus, the negative findings in this study cannot be explained from inadequate compliance alone, but most likely also suggest the exercise therapy program itself to be insufficient to engender gait alterations.

**Conclusions:**

Adding a 12-week supervised exercise therapy program to patient education did not induce changes in our selected biomechanical variables during the stance phase of gait, even when adjusting for poor compliance. Thus, we did not find evidence to support our exercise therapy program to be an efficacious intervention to induce gait alterations in this population of hip osteoarthritis patients.

**Trial registration:**

NCT00319423 at ClinicalTrials.gov (registration date 2006-04-26).

## Background

Hip osteoarthritis (OA) patients with mild to moderate symptoms, who are not yet candidates for total hip replacement (THR), may be considered candidates who would benefit from exercise therapy. However, as most studies regarding hip OA have included patients at a severe stage of disease, evidence for treatment modalities for this specific population of hip OA patients is limited. Gait abnormalities have been reported as one of the main clinical manifestations of hip OA [1,2]. A recent study from our research group reported the presence of distinct gait alterations at an early stage of disease; as hip OA patients revealed significantly reduced gait velocity, sagittal plane joint excursion and hip extension moment compared to healthy controls [3]. Studies examining whether gait biomechanics in early stage hip OA alter following exercise therapy interventions are of high clinical interest, due to the inherent potential of biomechanics as a target to impede disease progression [4]. However, no such studies exist. Thus, the aim of the present study was to compare gait in hip OA patients with mild to moderate symptoms participating in a randomized trial with two different interventions; patient education only or patient education followed by a 12-week supervised exercise therapy program. The main outcome of the overall randomized trail was self-reported pain, with gait included as one of several secondary outcome measures. At the time of study initiation, the existing knowledge on early stage hip OA gait was limited.Therefore, we did not consider excact hypotheses on specific treatment effects to be justified. Rather, our approach was explorative, based on a broad assessment of gait variables, and with no pre-defined hypotheses as to whether the exercise program utilized would influence gait pattern or not.

## Methods

## Material

This study was a biomechanical substudy of a larger randomized controlled trial (RCT) (Clinical Trials NCT00319423). The aim of the main RCT was to evaluate the efficacy of adding a supervised exercise therapy program to patient education, with self-reported pain assessed from The Western Ontario and McMaster Universities Arthritis Index (WOMAC) as primary outcome [5]. Patients aged between 40 and 80 years with uni- or bilateral hip pain for ≥3 months were eligible for participation. Inclusion criteria were symptomatic hip OA defined from the Harris Hip Score (HHS) [6], combined with radiographic OA verified by Danielsson’s criteria [7]. As an HHS <60 is a cut-off criteria for THR at our institution [8], and 100 reflects a perfect score, patients with an HHS <60 and ≥95 were excluded. Additional exclusion criteria were previous THR, knee pain, recent lower limb trauma and/or injury, neurological disorders, rheumatoid arthritis, cancer, heart disease, osteoporosis, low back pain, and/or inability to understand Norwegian.

**Figure 1:**
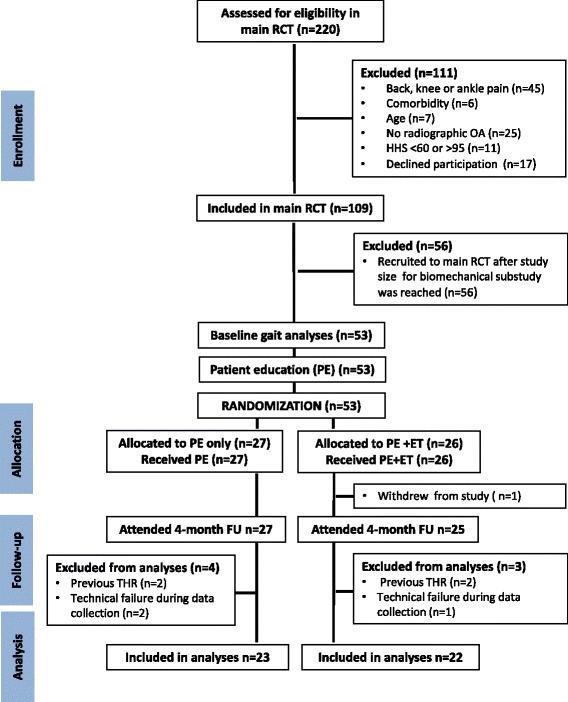
**Flow-chart of the study.** Abbreviations: RCT = randomized controlled trial, OA = osteoarthritis, HHS = Harris Hip Score, PE = patient education, ET = exercise therapy, FU = follow-up.

Power calculations based on WOMAC pain revealed a need for 109 patients in the main study. When conducting estimates for the biomechanical substudy, we were limited by two factors. Firstly, no previous studies could justify specification of a primary outcome measure for hip OA gait; and secondly, as a direct consequence, no cut-offs for minimal clinically important changes could be determined. We therefore based the biomechanical substudy power calculations on peak hip- and knee joint angles and moments from a previously conducted gait analysis study from our group, including patients with knee injuries [9]. Estimating a 10 % difference in knee- and hip joint angles in the sagittal and frontal plane between the groups at follow-up with an alpha level at 0.05, the number needed in each group, with 90 % test power, ranged from 16 to 21 patients for the different variables. Accounting for the highest estimated number, and a drop-out rate of 10 %, we decided to recruit the first consecutive 53 patients in the main RCT into the biomechanical substudy. Following a block randomization procedure with sealed envelopes, 27 out of the 53 patients were assigned to patient education only, and 26 assigned to patient education followed by a 12-week supervised exercise therapy program (Figure 1). Group allocation was blinded for all researchers involved in the biomechanical substudy. The analyses in this study are limited to the comparison of biomechanical outcome measures during the stance phase of gait between the two randomized groups. The primary outcome for the main RCT, WOMAC pain, as well as other clinical and performance-based outcome measures, have been reported in previous publications from our group [5,10]. Furthermore, comparisons of biomechanical characteristics of the hip OA patients to healthy controls during gait [3], and sit-to-stand [11], have also been described by our group, and are therefore not included in the present analyses.

All participants signed an informed consent before inclusion. The Regional Medical Research Ethics Committee of Eastern Norway gave study approval, and the study was conducted in agreement with the Helsinki Declaration.

### Interventions

The patient education was organized as three group sessions of a ‘hip school’, originally developed for hip OA patients by Klässbo et al. [12]. One of the intentions of the hip school, was to empower the participants to better manage pain, moderate impairments, and sustain their physical function. Each group session included six to seven patients, and lasted for approximately one hour. In addition, all patients had a one-to-one consultation with a physical therapist two months after completing the group sessions. The hip school started immediately after baseline testing.

A supervised exercise therapy program developed for hip OA patients was utilized for the exercise therapy intervention group [13]. The first session for each patient started within a week of completion of the hip school. The exercise therapy program comprised an initial warm-up procedure either on a treadmill or an ergometer cycle, followed by exercises targeted to improve muscle strength, physical function, neuromuscular control and flexibility. When walking on the treadmill, patients were instructed to emphasize equal cadence and to complete their ankle/toe push-off with an extended hip, but otherwise no specific gait exercises were included. A total number of 26 exercises was included in the program; of which patients conducted 8–12 exercises in each training session. Patients were instructed to always include a combination of exercises assuring that both muscle strength, physical function, neuromuscular control and flexibility were addressed. The dosage for strengthening exercises was three sets of eight repetitions at 70-80% of one repetition maximum (1RM), and for functional exercises three sets of 10 repetitions [13]. All patients were instructed to perform the exercise program two to three times per week. Individual supervision by a physical therapist specialized in orthopaedic and/or sports physical therapy was offered twice a week, of which one session was mandatory. During supervision, progression was customized for each individual patient. For strength exercises, resistance was increased when the patient could exceed eight repetitions, and for the functional exercises when the patient could exceed 10 controlled movement repetitions. Exercise was further regulated according to pain. Patients registered their training sessions in an exercise diary. The complete exercise program has been described in detail and is further available as an Appendix in a previous publication by Fernandes et al. [13].

### Subject characteristics

Pain duration, HHS, age, height and bodyweight were recorded at baseline. Body mass index (BMI) was calculated from the formula bodyweight/(height x height).

### Gait analysis

**Figure 2:**
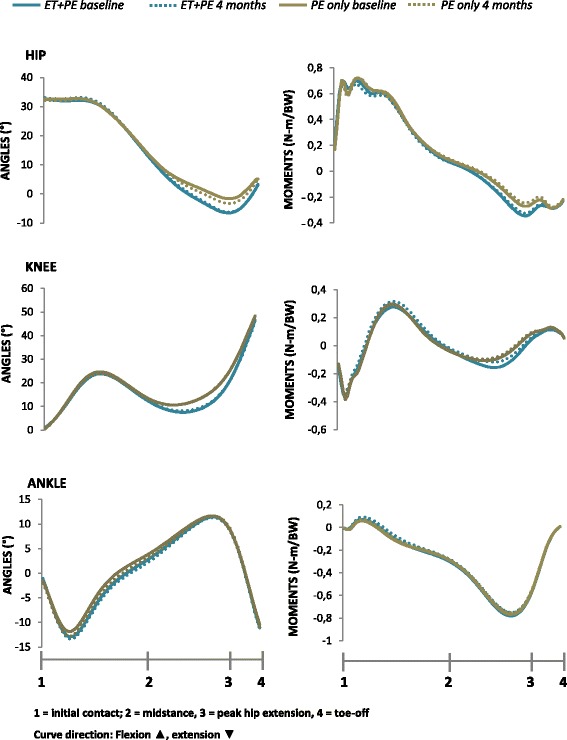
**Sagittal plane joint angle displacement and corresponding external moments during stance.** Abbreviations: PE + ET = Patient education + Exercise therapy (n = 22). PE only = Patient education only (n = 23).

**Figure 3:**
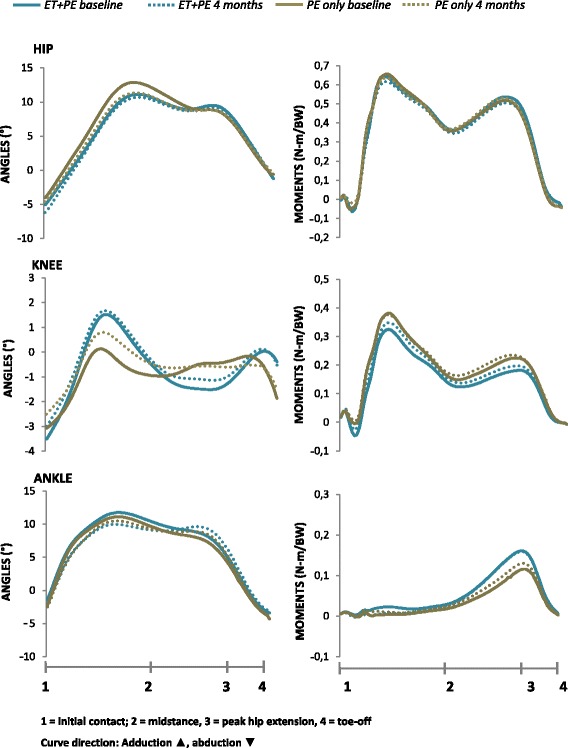
**Frontal plane joint angle displacement and corresponding external moments during stance.** Abbreviations: PE + ET = Patient education + Exercise therapy (n = 22). PE only = Patient education only (n = 23).

Gait analyses were conducted at the motion analysis lab at The Norwegian School of Sport Sciences, at baseline and at a four month follow-up; when the participants in the exercise therapy group had completed their program. A Qualisys pro-reflex motion analysis system (Qualisys AB, Gothenburg, Sweden) with eight cameras was synchronized with two AMTI LG6 force plates (Advanced Mechanical Technology Inc, Watertown, MA, US) embedded in the floor. Sampling frequencies were 240 Hz for kinematic data and 960 Hz for kinetic data. The lower limb joint centers were defined by bilaterally placing reflective passive markers over anatomical landmarks: The medial and lateral malleolus, medial and lateral femoral condyle, the greater trochanter, and the top iliac crest. Additionally, three reflective passive markers rigidly attached on thermoplastic shells were placed at the sacrum and at the thigh and shank of both legs, and feet were defined bilaterally by two heel markers and one marker at the 5^th^ metatarsal head. Patients were instructed to look straightforward and walk at their self-selected speed along a 17-meter walkway. Photoelectric beams located 3.06 m apart, midway along the walkway, measured velocity. Ten of the included patients had bilateral hip OA. However, no significant systematic differences were found in any biomechanical variables between these patients and the patients with unilateral involvement. Therefore, only the target limb (defined as the most painful hip joint) was included in the analyses. Laroche et al. [14] have previously suggested five to 10 complete trials to be required to assure adequate reliability in hip OA gait analysis. Thus, we continued trials until we accomplished 12 satisfactory strikes for the target limb on the force plates. Of these, we selected six to eight trials within ±5% of the average velocity to be included for each subject, and calculated the mean value for each dependent variable. In the analyses, we utilized the mean values of all subjects. The mean of all subjects is also underlying the ensemble average curves shown in Figures 2 and 3.

Data were processed with Visual 3D software (C- motion Inc, Crabbs Branch Way Rockville MD). The stance phase of gait was normalized to 100% from initial contact to toe-off. We further defined the following events during stance: Initial contact (threshold 25 N), midstance (identified as the midpoint temporal observation of the stance phase when normalized from 0-100%), peak hip extension (peak hip extension angle) and toe-off (threshold 25 N) [3]. Sagittal and frontal plane joint angles (°) and external moments in Newton-meters normalized to bodyweight (N-m/BW) for the hip, knee, and ankle were calculated at each event.

### Radiographic assessment

The minimal joint space (MJS) in millimeters (mm) of the target hip joint was measured on standardized postero-anterior digital pelvic radiographs (Syngo Imaging V36, Siemens AG, Erlangen, Germany), centered on the symphysis.

### Statistical analyses

To compare the effectiveness of adding the 12-week supervised exercise therapy program to patient education, a one-way between-groups of analysis of covariance (ANCOVA) was conducted. The analysis model was built with group allocation as the independent variable, the four month post-test sagittal and frontal plane joint angles and moments of the hip, knee and ankle at the four selected events during stance as the dependent variables, and the corresponding baseline scores as covariates. Prior to the analysis, we made sure that the specific assumptions for normality and homeogeneity of variance for the one-way ANCOVA were met. Adherence to the exercise program was calculated as the median (inter-quartile range; IQR) of the number of completed sessions. As a supplementary analysis, the association between the number of completed exercise sessions and change in each of the kinematic or kinetic variables was calculated. As several of the change scores were not normally distributed, the Spearman’s rank correlation coefficient was chosen. Significance level was set to p < 0.05, and all analyses conducted in SPSS 18.0 (SPSS Inc., Chicago, IL, US).

## Results

All the 53 included patients completed gait analysis data collection at baseline. However, four of the 53 had gone through previous unilateral THR surgery. These patients were eligible for the main RCT, but were not intended to participate in the biomechanical substudy. Thus, they were excluded from the material. Furthermore, three patients were excluded due to incomplete data/technical failure at baseline (n = 1) or follow-up (n = 2), and finally one subject withdrew from participation before the four month follow-up. The final material thus consisted of 45 patients; 23 in the patient education only group, and 22 in the patient education + exercise therapy group (Figure 1 and Table 1).

**Table 1:** **Subject characteristics at baseline**

	**Patient education only (n = 23)**	**Patient education + exercise therapy (n = 22)**
	***Mean (SD)***	***Mean (SD)***
**Sex (males/females)**	10/13	9/13
**Age (years)**	58.2 (10.02)	60.2 (9.49)
**Pain duration (months)**	43.6 (36.60)	52.4 (65.75)
**Target joint (left/right)**	14/9	10/12
**Minimal joint space (mm)**	1.76 (1.057)	2.03 (1.092)
**Body mass index**	25.4 (3.81)	24.3 (2.19)
**Harris hip score**	75.3 (7.62)	80.7 (7.58)

All patients completed their three scheduled sessions of patient education. The median (IQR) number of completed exercise therapy sessions in the exercise therapy group was 19 (24–15); which implies a mean of 1.6 sessions per week. Only nine out of the 22 patients met the compliance criteria of ≥24 training sessions, specified in our clinical trials-registered protocol. A supplementary correlation analysis between the number of exercise therapy sessions and change in each of the 48 defined biomechanical variables revealed neglible to weak associations only, with Spearman’s rank values ranging from −0.007 to −0.383 (negative) and 0.045 to 0.324 (positive). None of the associations were statistically significant (p-values ranging from 0.093 to 0.974), and the direction of the associations appeared to be arbitrary. Thus, we did not find any support for larger changes in the dependent variables for the subjects who met the compliance criteria, than for those who did not.

The mean (SD) gait velocity at baseline was 1.51 (0.155) and 1.53 (0.134) meters per second, for the patient education only and patient education + exercise therapy group, respectively. At follow-up, corresponding values were 1.50 (0.172) and 1.52 (0.149). No statistical difference was found between the groups at follow-up (p = 0.827, partial eta squared .001). No significant differences were observed between the groups in joint angles or moments at the four month follow-up (Figures 2 and 3, Tables 2 and 3). The corresponding partial eta square values were all <0.02 for the joint angle variables (Table 2) and <0.09 for the joint moment variables (Table 3).

**Table 2:** **Joint angles; baseline and 4-month follow-up between groups: ANCOVA with baseline as covariate**

		**Patient education only (n = 23)**			**Patient education + exercise therapy (n = 22)**			**P**	**Part. Eta sq.**
		**Baseline**	**Follow-up**	**Change**	**Baseline**	**Follow-up**	**Change**		
		***Mean (SD)***			***Mean (SD)***				
**Hip**									
Sagittal plane	Initial contact	32.1 (4.24)	32.7 (6.03)	0.6 (5.10)	32.6 (5.29)	33.0 (5.26)	0.4 (4.61)	.969	.000
	Midstance	13.2 (4.18)	13.1 (4.61)	−0.1 (5.48)	12.5 (6.59)	12.7 (4.53)	0.2 (5.27)	.874	.001
	Peak hip extension	−3.4 (5.93)	−2.4 (7.31)	1.0 (6.09)	−6.7 (9.13)	−5.7 (7.27)	1.0 (5.13)	.545	.009
	Toe-off	5.3 (5.53)	5.1 (6.69)	0.2 (6.82)	3.2 (8.38)	3.7 (6.60)	0.5 (6.42)	.857	.001
Frontal plane	Initial contact	−4.0 (4.35)	−4.6 (4.78)	−0.6 (4.59)	−5.1 (3.85)	−6.2 (3.88)	−1.1 (2.99)	.392	.018
	Midstance	11.7 (3.48)	10.5 (3.54)	−1.2 (3.72)	10.4 (4.11)	10.1 (4.02)	−0.3 (2.44)	.606	.006
	Peak hip extension	5.3 (4.16)	5.4 (3.79)	0.1 (2.21)	6.0 (4.55)	5.9 (3.62)	−0.1 (3.08)	.931	.000
	Toe-off	−0.6 (3.25)	−0.5 (3.19)	0.1 (2.50)	−1.2 (3.73)	0.9 (3.17)	0.3 (3.03)	.887	.001
**Knee**									
Sagittal plane	Initial contact	0.8 (3.65)	0.5 (3.70)	−0.3 (3.20)	1.2 (4.75)	1.0 (3.37)	−0.2 (3.50)	.770	.002
	Midstance	13.3 (4.40)	13.0 (4.45)	−0.3 (2.79)	12.3 (5.50)	12.3 (4.40)	0.0 (2.79)	.989	.000
	Peak hip extension	22.6 (7.06)	22.8 (7.59)	0.2 (2.77)	18.2 (6.52)	18.2 (6.45)	0.0 (3.27)	.665	.005
	Toe-off	48.4 (5.11)	47.9 (5.66)	−0.5 (2.99)	46.5 (5.63)	45.7 (5.76)	−0.8 (3.63)	.616	.006
Frontal plane	Initial contact	−3.1 (3.04)	−2.5 (3.18)	0.6 (1.96)	−3.5 (2.38)	−3.0 (3.16)	0.5 (1.75)	.893	.000
	Midstance	−1.0 (3.32)	−0.6 (2.94)	0.4 (2.42)	−0.8 (2.84)	−0.6 (3.52)	0.2 (2.11)	.803	.002
	Peak hip extension	−0.2 (4.06)	−0.5 (3.39)	−0.3 (2.50)	−0.6 (3.82)	−0.4 (4.07)	0.2 (2.80)	.543	.009
	Toe-off	−1.9 (8.49)	−1.5 (6.74)	0.4 (4.70)	−0.4 (7.90)	−0.5 (6.81)	−0.1 (5.81)	.979	.002
**Ankle**									
Sagittal plane	Initial contact	−1.6 (3.60)	−2.1 (4.10)	−0.5 (3.44)	−1.2 (3.35)	−1.1 (3.18)	0.1 (2.08)	.392	.019
	Midstance	4.0 (2.79)	3.3 (2.59)	−0.7 (2.19)	3.0 (3.22)	2.4 (2.99)	−0.6 (2.31)	.633	.006
	Peak hip extension	9.0 (2.92)	9.3 (2.79)	0.3 (1.93)	8.9 (4.51)	8.7 (3.89)	−0.2 (3.01)	.445	.015
	Toe-off	−10.4 (4.07)	−11.0 (4.20)	−0.6 (2.78)	−11.1 (5.44)	−11.6 (5.75)		.954	.000
Frontal plane	Initial contact	2.1 (3.99)	2.5 (3.97)	0.4 (2.68)	2.3 (3.44)	1.4 (4.30)	0.9 (3.21)	.835	.001
	Midstance	−9.0 (2.48)	−9.2 (4.25)	−0.2 (3.05)	−10.1 (2.67)	−9.3 (2.19)	0.8 (2.58)	.612	.006
	Peak hip extension	−3.2 (3.76)	−2.5 (4.25)	0.7 (3.54)	−2.4 (4.33)	−1.4 (3.75)	1.0 (3.33)	.582	.008
	Toe-off	3.2 (4.57)	3.5 (4.25)	0.3 (3.71)	3.4 (5.36)	4.3 (4.51)	0.9 (3.32)	.583	.008

SD = standard deviation.

P = p-value.

Part. Eta sq. = partial eta squared.

Mean scores for the two groups are shown in columns *Baseline* and *Follow-up* and the change from baseline to follow-up in the columns *Change*. The results of the ANCOVA (statistical test of the difference between the groups at follow-up, adjusted for score at baseline) is revealed in columns *P* (significance level)*,* and *Partial Eta squared* (effect size).

**Table 3:** **Joint moments; baseline and 4 month follow-up between groups: ANCOVA with baseline as covariate**

		**Patient education only (n = 23)**			**Patient education + exercise the rapy (n = 22)**			**P**	**Part. Eta sq.**
		**Baseline**	**Follow-up**	**Change**	**Baseline**	**Follow-up**	**Change**		
		***Mean (SD)***			***Mean (SD)***				
**Hip**									
Sagittal plane	Initial contact	-.166 (.679)	-.181 (.065)	-.015 (.048)	-.208 (.093)	-.236 (.114)	-.028 (.076)	.385	.020
	Midstance	-.097 (.070	-.095 (.080)	.002 (.056)	-.081 (.064)	-.077 (.063)	.004 (.073)	.298	.028
	Peak hip extension	.258 (.118)	.284 (.106)	.026 (.076)	.325 (.102)	.309 (.101)	-.017 (.090)	.178	.047
	Toe-off	.218 (.064)	.229 (.058)	.011 (.035)	.231 (.042)	.231 (.041)	.000 (.031)	.943	.000
Frontal plane	Initial contact	-.007 (.043)	-.007 (.030)	.000 (.040)	.004 (.050)	-.007 (.051)	-.011 (.030)	.726	.003
	Midstance	-.359 (.093)	-.368 (.090)	-.009 (.047)	-.366 (.097)	-.350 (.092)	.016 (.061)	.106	.065
	Peak hip extension	-.313 (.130)	-.316 (.114)	-.003 (.067)	-.357 (.095)	-.329 (.076)	.028 (.095)	.557	.009
	Toe-off	.043 (.035)	.044 (.024)	-.001 (.027)	.033 (.038)	.041 (.037)	.008 (.033)	.707	.004
**Knee**									
Sagittal plane	Initial contact	-.136 (.035)	-.130 (.028)	.006 (.029)	-.154 (.056)	-.164 (.062)	-.010 (.040)	.063	.086
	Midstance	-.052 (.085)	-.026 (.061)	.026 (.099)	-.029 (.080)	-.016 (.059)	.013 (.069)	.751	.003
	Peak hip extension	.108 (.099)	.090 (.080)	.018 (.077)	.052 (.080)	.064 (.058)	.012 (.085)	.276	.030
	Toe-off	.054 (.025)	.056 (.026)	.002 (.156)	.057 (.017)	.056 (.019)	.001 (.020)	.646	.005
Frontal plane	Initial contact	-.019 (.020)	-.022 (.017)	-.003 (.020)	-.023 (.022)	-.021 (.023)	-.002 (.019)	.630	.007
	Midstance	-.144 (.058)	-.171 (.069)	-.027 (.055)	-.137 (.066)	-.139 (.079)	-.002 (.046)	.120	.066
	Peak hip extension	-.158 (.080)	-.169 (.069)	-.011 (.058)	-.142 (.073)	-.146 (.078)	-.004 (.071)	.466	.015
	Toe-off	.006 (.015)	.008 (.013)	.002 (.013)	.007 (.018)	.011 (.017)	.004 (.016)	.503	.013
**Ankle**									
Sagittal plane	Initial contact	-.004 (.008)	-.004 (.008)	.000 (.008)	-.006 (.012)	-.008 (.016)	-.002 (.012)	.439	.015
	Midstance	-.291 (.060)	-.286 (.086)	.005 (.067)	-.302 (.066)	-.281 (.081)	.021 (.087)	.584	.008
	Peak hip extension	-.601 (.083)	-.590 (.103)	.011 (.066)	-.609 (.074)	-.589 (.061)	.020 (.095)	.810	.002
	Toe-off	.009 (.009)	.006 (.009)	-.003 (.006)	.009 (.006)	.008 (.010)	-.001 (.007)	.144	.054
Frontal plane	Initial contact	-.006 (.004)	-.006 (.003)	.000 (.004)	-.006 (.004)	-.009 (.013)	-.003 (.128)	.243	.034
	Midstance	-.018 (.033)	-.025 (.040)	-.007 (.038)	-.029 (.033)	-.026 (.034)	.003 (.032)	.623	.006
	Peak hip extension	-.113 (.057)	-.126 (.068)	-.013 (.058)	-.153 (.050)	-.151 (.060)	.002 (.055)	.970	.000
	Toe-off	-.002 (.004)	-.004 (.004)	-.002 (.003)	-.005 (.005)	-.005 (.006)	.000 (.005)	.210	.039

SD = standard deviation.

P = p-value.

Part. Eta sq. = partial eta squared.

Mean scores for the two groups are shown in columns *Baseline* and *Follow-up* and the change from baseline to follow-up in the columns *Change*. The results of the ANCOVA (statistical test of the difference between the groups at follow-up, adjusted for score at baseline) is revealed in columns *P* (significance level)*,* and *Partial Eta squared* (effect size).

## Discussion

We found no differences in either gait velocity or stance phase sagittal and frontal plane joint angles or moments between patients who had received patient education only and patients who had conducted a 12-week supervised exercise therapy program in addition to patient education. Partial effect sizes overall revealed < 9% of the variance in outcome at post-test to be explained by group allocation. Hence, interventions did not appear to cause any evident alterations in gait in either group.

The established position of exercise therapy as a core first-line treatment in OA management has recently been confirmed in a meta-analysis by Uthman et al. [15], and also in updated guidelines from the European League Against Rheumatism (EULAR) [16], the Osteoarthritis Research Society International (OARSI) [17] and the American College of Rheumatism (ACR) [18]. As the exercise therapy program utilized in this study comprised multiple exercises targeting muscle strength, physical function, neuromuscular control and flexibility, its content was in accordance with current recommendations. However, whereas compliance to the patient education was 100%, compliance to the exercise therapy program was insufficient, with only nine patients accomplishing ≥24 sessions. Unfortunately, as reasons for inadequate adherence to the required number of sessions were not registered in the training diaries, they are not fully known. However, only one patient discontinued due to increased hip pain [5]. The lack of treatment effects on gait could be reflecting lack of adequate participation, rather than lack of efficacy of the program itself. Since per-protocol analyses would be underpowered, we conducted a supplementary correlation analysis to assess any association between the number of completed exercise therapy sessions and changes in gait. The results did, however, not suggest any beneficial effect of the exercise therapy program even for those compliant. It is, thus, plausible to suggest that the program itself also may have been inadequate to engender alterations in gait assessed by our selected kinematic and kinetic variables. In particular, we did not find any improvements in hip- and knee joint extension and the accompanying hip moment; the variables previously shown to be most deviant compared to age-matched healthy subjects [3]. A larger study sample may, however, be required in future studies in order to provide robust findings as to whether improved adherence may influence gait. As we did not include electromyographic (EMG) assessments, we do not know whether the exercise program may have induced any neuromuscular alterations, which were not reflected as altered joint angles and moments. Furthermore, as this study is a substudy of a larger trial; self-reported and performance-based outcome measures were not included. Previous reports on the overall RCT have shown improvements in WOMAC physical function, but not pain, in the exercise therapy intervention group [5,10]. Results on performance based assessments have not yet been published.

The exercise therapy program did not include specific gait modification approaches or instructions for each individual patient. As our study is the first to report data based on a randomized study to investigate effects of exercise therapy on gait in early stage hip OA, we cannot compare our findings to any analogous cohorts. Several investigations have, however, evaluated whether exercise therapy alter gait in patients with early stage knee OA [19-23]. None of these studies reported evidence for adaptations in joint loading after muscle strengthening exercises targeting quadriceps and/or hip abductor muscles, despite improvements in muscle strength and/or self-reported symptoms. In contrast to our exploratory approach, it must be noted that the majority of these studies primarily focused on the peak knee adduction moment. Our findings do *not* support generalized exercise therapy programs to be efficacious in reversing gait adaptations, even if current evidence confirms the presence of gait alterations in early stage lower limb OA. However, divergence between the demands posed to the joints and muscles during the exercises included in the protocol, and during the outcome measure *gait*, may represent a limitation. We cannot rule out possible improvements in muscle strength and/or neuromuscular control and balance, that were not reflected in the emergent joint angles or moments. As stated by Winter [24], emergent joint angles may stem from a broad range of moment of force patterns. The inherent within-subject variability in human movement may, thus, diminish our ability to detect robust group level changes when investigating exercise therapy interventions from a biomechanical context. This assumption is true even when looking into existing studies on targeted gait interventions. A recent review by Khalaj et al. [25] suggested specific gait retraining programs to be advantageous to reduce knee adduction moment in patients with knee OA, whereas the efficacy of more generalized exercise programs was found to be inconclusive. In contrast, the findings reported in the systematic review by Simic et al. [26] were inconclusive regarding the efficacy of targeted gait modification strategies to alter knee joint loads. Future studies addressing specific, tailored exercises intended to alter evident gait deviations in early stage hip OA are thus warranted.

There is currently limited evidence defining the optimal exercise program and the ideal dosage for lower limb OA patients. Our exercise therapy program was developed in line with current recommendations at the time [13]. However, it has been argued that existing protocols have been both of too short duration and low intesity/load; potentially because of the concern that intensive training could worsen symptoms [27]. In a recent meta-analysis, Juhl et al. [28] found larger pain reduction in patients who conducted frequent supervised exercise therapy sessions, and consequently recommend as many as three supervised weekly sessions for best efficacy. It is not possible from existing studies to estimate the required dosage and intensity needed to induce changes in gait, and the required dosage needed for gait changes to be clinically meaningful [26]. Hagen et al. [29] concluded in a recent meta-analysis that there is an evident knowledge gap in our understanding of the mechanisms by which the potential effect of exercise therapy occurs in musculoskeletal disorders; including OA. As previously mentioned, the optimal dosage and frequency of exercise is not known, nor is the specific components that should be included in exercise programs to customize interventions for different conditions. In their systematic review, Bennell and Hinman [30] support these notions, by stating that the known effects of exercise on structural disease progression is sparse. Consequently, our knowledge is still limited on how exercise therapy may influence disease pathogenesis and possibly prevent or slow down disease progression. It is, however, interesting to note that despite a lack of short-term improvements, our research group found the need for THR to be reduced in the exercise therapy intervention group in a long-term follow-up of the main RCT recently published by Svege et al. [10]. Furthermore, the patients in the exercise therapy group reported better scores in self-reported physical function. These long-term effects are of definitive clinical interest, however, the underlying explanations for a possible slower symptom progression are unclear and require further investigation. This notion is true also from a biomechanical context. Whereas adequate mechanical loading is a vital stimulus for joint homeostasis; cumulative stress caused by abnormal joint loading conversely may have a negative influence on joint deterioration and disease progression in lower limb OA [4,31-33]. However, the contribution of specific biomechanical factors remains unclear [34]. Many existing gait analyses are underpowered [35], whilst there are few studies evaluating hip OA compared to knee OA. In addition, as seen in this study, gait variables typically reveal large standard deviations reflecting considerable dispersion in data. Furthermore, three recent meta-analyses by Ewen et al. [36], Constantinou et al. [37] and Mills et al. [38], all emphasized the vast diversity in reported outcome measures in existing studies examining gait in OA. This lack of consensus makes it difficult to synthesize existing knowledge into reasonable hypotheses, and to define which specific gait variables should be targeted during exercise therapy and/or gait modification programs.

### Study limitations

This study is the first to report the effects of an exercise intervention on gait in hip OA patients with mild to moderate symptoms from a randomized design. In contrast to the majority of hip OA gait studies, our sample size was based on a priori power calculations, and the number of patients included in the final material were in accordance with the estimated study size. It must, however, be regarded as a limitation that the biomechanical gait variables reported in this study were secondary outcome measures from a larger, randomized trial, using WOMAC pain as primary outcome. The minimal clinically relevant change in each of the selected gait parameters could not be accurately decided when the study was initiated, and is still uncertain today. Thus, our sample size calculations may not have been precise enough to assure an adequate study power, and the apparent lack of treatment effects must be interpreted within this context. Another limitation is that the low compliance in the exercise therapy group was inadequate to realize the potential inherent in the randomized design. The results should therefore be considered as explorative rather than conclusive. This precaution is reinforced by the evident diversity in methods and outcome mesaures in existing hip OA gait studies; which diminishes our ability to evaluate the external validity of our findings.

## Conclusions

We found no significant effects of a generalized 12-week exercise therapy program for sagittal or frontal plane lower extremity joint angle displacement or moments during the stance phase of gait in hip OA patients with mild to moderate symptoms, even when adjusting for poor compliance. Thus, we did not find evidence to support our exercise therapy program as an efficacious intervention to induce gait alterations in this population of hip OA.

## Abbreviations

OA: Osteoarthritis
RCT: Randomized controlled trial
HHS: Harris hip score
THR: Total hip replacement
WOMAC: The Western Ontario and McMaster Universities arthritis index
N-m: Newton-meter
BW: Bodyweight
MJS: Minimal joint space
IQR: Interquartile range
SD: Standard deviation
EULAR: European league against rheumatism
OARSI: Osteoarthritis research society international
ACR: American college of rheumatism
